# Tracking longitudinal thalamic volume changes during early stages of SCA1 and SCA2

**DOI:** 10.1007/s11547-024-01839-2

**Published:** 2024-07-02

**Authors:** Marina Grisoli, Anna Nigri, Jean Paul Medina Carrion, Sara Palermo, Greta Demichelis, Chiara Giacosa, Alessia Mongelli, Mario Fichera, Lorenzo Nanetti, Caterina Mariotti

**Affiliations:** 1 Neuroradiology Unit, Fondazione IRCCS Istituto Neurologico Carlo Besta, Via Celoria 11, Milan, Italy; 2 Department of Psychology, University of Turin, Turin, Italy; 3 Unit of Medical Genetics and Neurogenetics, Fondazione IRCCS Istituto Neurologico Carlo Besta, Milan, Italy

**Keywords:** Thalamus, Presymptomatic carriers, Spinocerebellar ataxias, MRI, SCA1, SCA2

## Abstract

**Purpose:**

Spinocerebellar ataxia SCA1 and SCA2 are adult-onset hereditary disorders, due to triplet CAG expansion in their respective causative genes. The pathophysiology of SCA1 and SCA2 suggests alterations of cerebello-thalamo-cortical pathway and its connections to the basal ganglia. In this framework, thalamic integrity is crucial for shaping efficient whole-brain dynamics and functions. The aims of the study are to identify structural changes in thalamic nuclei in presymptomatic and symptomatic SCA1 and SCA2 patients and to assess disease progression within a 1-year interval.

**Material and methods:**

A prospective 1-year clinical and MRI assessment was conducted in 27 presymptomatic and 23 clinically manifest mutation carriers for SCA1 and SCA2 expansions. Cross-sectional and longitudinal changes of thalamic nuclei volume were investigated in SCA1 and SCA2 individuals and in healthy participants (n = 20).

**Results:**

Both SCA1 and SCA2 patients had significant atrophy in the majority of thalamic nuclei, except for the posterior and partly medial nuclei. The 1-year longitudinal evaluation showed a specific pattern of atrophy in ventral and posterior thalamus, detectable even at the presymptomatic stage of the disease.

**Conclusion:**

For the first time in vivo, our exploratory study has shown that different thalamic nuclei are involved at different stages of the degenerative process in both SCA1 and SCA2. It is therefore possible that thalamic alterations might significantly contribute to the progression of the disease years before overt clinical manifestations occur.

**Supplementary Information:**

The online version contains supplementary material available at 10.1007/s11547-024-01839-2.

## Introduction

Spinocerebellar ataxia type 1 (SCA1, MIM 164,400) and type 2 (SCA2, OMIM 183090) are autosomal dominant inherited diseases caused by an expansion of a highly polymorphic CAG repeat sequence within the coding region of the *ATXN1* and *ATXN2* gene, respectively. The typical clinical presentation is a progressive cerebellar syndrome with adult onset [1]. Gait difficulties or other cerebellar symptoms mark the transition from presymptomatic to symptomatic SCA individuals [2]. Although gait ataxia defines the clinical onset of the disease, presymptomatic individuals often present with a wide range of subtle cerebellar and non-cerebellar symptoms, including diplopia, dysarthria, episodic vertigo, handwriting difficulties, and sensory and sleep disturbances [2–4]. Understanding early pathophysiological changes in the preclinical phase is crucial to advance our knowledge of the SCA pathogenesis and identify reliable biomarkers to follow disease progression [2].

Neuroimaging studies in SCA1 and SCA2 have revealed distinct patterns of grey and white matter alterations, mainly involving the cerebellum and brainstem [5, 6]. Yet, regional brain structural alterations, that extend past the cerebellum and spread to cortical and subcortical regions, have been observed in both presymptomatic and symptomatic SCA1 and SCA2 patients [7–15]. Post-mortem neuropathological studies have confirmed extensive neurodegeneration of the thalamic subnuclei—localized mainly in a subset of the ventral basal nuclei—thus providing an anatomical explanation for some of the disease-related symptoms in SCA1 [16] and SCA2 [17, 18] patients at different stages of the disease. In support of this hypothesis, it has been observed that deep brain stimulation of the ventral intermediate nucleus of the thalamus and subthalamic nucleus improves severe tremor in patients with SCA2, by acting on the cerebello-thalamo-cortical circuit [19, 20].

Thus, the pathophysiology of spinocerebellar ataxia could involve the alteration of a broader circuit that includes the cerebello-thalamo-cortical pathway and its connections to the basal ganglia since the presymptomatic phase [6, 21, 22]. As the thalamus acts not only as a relay station, but as a hub integrating information among cerebellar, cortical, and subcortical areas, thalamic integrity is crucial in shaping the dynamics of the whole brain [23, 24]. Indeed, previous findings reported significant structural alterations [14, 15, 25–30] and functional organization changes in the thalamus [31] in both SCA1 and SCA2 patients.

In vivo assessment of thalamic nuclei volume by MRI could therefore prove to be a powerful approach if the structural spread of disease in SCA1 and SCA2 patients is to be tracked from the preclinical stages of the disease. To the best of our knowledge, there are no studies characterizing the in vivo structural alterations of thalamic nuclei in symptomatic SCA1 and SCA2 patients. Importantly, no characterization has been attempted in presymptomatic subjects.

Our main aims were to (1) describe in vivo structural changes in thalamic nuclei in presymptomatic and symptomatic SCA1 and SCA2 patients in comparison with healthy subjects and (2) verify whether a 1-year longitudinal interval will allow quantification of disease progression changes in these subnuclei. We here hypothesized specific early abnormalities of the ventral thalamic nuclei, mainly targeting the cerebellar-thalamo-cortical and the basal ganglia-thalamo-cortical pathways [22, 32], prior to the onset of clinical symptoms in pre-manifest subjects and—later—associated with disease progression in symptomatic patients.

## Material and methods

### Participants

Between January 2015 and July 2017, our centre specialized in spinocerebellar ataxia examined 50 adult subjects from families with SCA1 and SCA2. We enrolled 10 ataxic patients and 14 presymptomatic SCA1 mutation carriers (preSCA1), and 13 ataxic patients and 13 presymptomatic SCA2 mutation carriers (preSCA2). We also include in the study 20 family members (CTRL), who were SCA1 and SCA2 gene-negative. The complete characteristics of the participants have been previously reported [14, 15, 33]. The presence and severity of ataxia was evaluated using the Scale for Assessment and Rating of Ataxia (SARA) [34]. For SCA1 and SCA2 patients, the disease duration was calculated as the difference between age at enrolment and age of ataxia manifestation, while for presymptomatic carriers, the estimated years-to-disease onset was based on CAG repeat length [35]. All clinical and neuroimaging investigations were conducted blindly with respect to the genetic status. The study was approved by the Ethics Committee of Regione Lombardia Sezione Fondazione IRCCS Istituto Neurologico “Carlo Besta”, and written informed consent was obtained for all participants.

### MRI acquisition and analyses

MRI scans were performed on all participants at baseline and 1-year follow-up, using a 3 T scanner (Achieva, Philips Healthcare NL) equipped with a 32-channel cranial coil. T1-weighted (T1w) high-resolution 3D MRI (repetition time = 9.781 ms, echo time = 4.6 ms, field of view = 240 × 240 mm, no gap, voxel size = 1 × 1 × 1 mm, flip angle = 8°, 185 sagittal slices), T2-weighted axial T2-weighted turbo spin echo sequences, and 3D fluid-attenuated inversion recovery were acquired in each MRI scan. Neuroradiologists examined the images for standard diagnostic purposes and to rule out incidental findings in study participants.

Thalamic nuclei volume analysis was conducted on T1w images acquired at baseline and follow-up MRI scans. To assess the volumes of the subnuclei of the thalamus, FreeSurfer software (http://freesurfer.net, version 7) was used. For each participant, N4 bias field correction was applied as a preprocessing step on T1w image to normalize variations in image intensity across the volume [36]. T1w images were processed through the “recon-all” pipeline. Then, the thalamic nuclei segmentation module [37] was applied. After a visual check of the segmentation obtained for each thalamic nucleus, no manual intervention was performed on the data. The volume of each thalamic subnucleus was extracted. The total intracranial volume (TIV) was obtained from the “aparc” file. To limit the number of comparisons, the 25 thalamic nuclei for each hemisphere were grouped into 14 sub-regions according to [38]; then, the left and the right nuclei were summed: anteroventral (AV), laterodorsal (LD), lateroposterior (LP), anterior ventral (VA), anterior lateral ventral (VLa), posterior lateral ventral (VLp), posterolateral ventral (VPL), ventromedial (VM), intralaminar, midline, mediodorsal (MD), lateral geniculate (LGN) and medial geniculate (MGN), pulvinar. [The association between the FreeSurfer parcellation and these sub-regions is shown in Supplementary Table 1 according to [38].] All thalamic subnuclei volumes were expressed as a percentage of the TIV [39].

### Statistical analyses

For each bilateral thalamic nucleus, the median percentage difference between each pathological group and the CTRL was computed.

To identify cross-sectional differences in thalamic subnuclei volumes between groups (CTRL, preSCA1, SCA1 participants; CTRL, preSCA2, and SCA2 participants), multinomial logistic regression was applied separately at baseline and at follow-up, revealing areas that clearly distinguished groups from each other.

To observe longitudinal changes in thalamic nuclei volume between baseline and follow-up evaluation within each pathological group (preSCA1, SCA1, preSCA2, SCA2), the Wilcoxon signed rank test was used. The longitudinal percent difference was obtained for each thalamic nuclei as percentage of the median variation between baseline and 1-year follow-up within each group [15].

The standardized response mean was reported as longitudinal effect size index for the ventral thalamic portion including the VPL, VLa, VLp nuclei [9]. Specifically, this portion of the thalamus was chosen because it is reported to be the most common targets of the degenerative process in both SCA1 and SCA2 [16, 18] and to have the greatest connection strength for the dentate-thalamic projections [32]. Values of 0.2, 0.5, 0.8, 1.2, and 2.0 were considered small, medium, large, very large, and huge changes [40].

The baseline volume of the portion including the VPL, VLa, VLp nuclei was correlated with SARA score (Spearman correlation) and time of disease progression (Pearson correlation). Time was analysed as a single variable, taking into account the duration of the disease (the gap between enrolment age and onset age) for symptomatic patients and years before onset for presymptomatic subjects (method by [35]). To assess whether there is a correlation between cerebellar and thalamic degeneration, a Pearson correlation was applied. The volume of this ventral thalamic portion at baseline was also correlated with total cerebellar volume and the sum of cerebellar lobules with the most severe degree of atrophy in SCA1 and SCA2 (i.e. IV, V, VI, Crus II, VIIB, VIIIA, VIIIB for SCA1 compared to CTRL, defined as cluster 1 in [15]; lobules I–II, IV, VI for SCA2 compared to CTRL in [14]). All statistical analyses were conducted using R software version 4.0.3 (2020–10-10) [41].

## Results

### Clinical assessments

Participants’ baseline and 1-year follow-up characteristics are summarized in Table 1. There was a significant difference between preSCA1 and CTRL in terms of median age (*p* < 0.01). At 1-year follow-up, 53 subjects (76%) underwent longitudinal assessments: 9 SCA1 patients (90%), 8 preSCA1 (57%), 13 SCA2 patients (100%), 9 preSCA2 (69%), and 14 CTRL (70%).

**Table 1:** Clinical characteristics of participants

	Baseline			1-year follow-up		
	CTRL	preSCA1	SCA1	CTRL	preSCA1	SCA1
N	20	14	10	14	8	9
Gender (Female/Male)	15/5	5/9	2/8	11/3	3/5	2/7
Age (years)	29 (19–48)	29 (18–50)	44 (33–51) **	33 (20–51)	38 (21–51)	45 (35–51) *
Years from onset	–	− 9.72 (− 32.30 | − 3.52)	–	–	− 6.29 (− 11.02 | − 3.52)	–
Disease duration after onset (years)	–	–	6.28 (2.13 | 13.23)	–	–	6.89 (3.42 | 17.73)
SARA score	0 (0–2)	0.5 (0–2) **	6.5 (2.5–20) ***			

	CTRL	preSCA2	SCA2	CTRL	preSCA2	SCA2
N	20	13	13	14	9	13
Gender (Female/Male)	15/5	9/4	4/9	11/3	6/3	4/9
Age (years)	29 (19–48)	40 (19–50)	35 (28–50)	33 (20–51)	41 (21–52)	36 (30–51)
Years from onset	–	− 15.5 (− 30.9 | − 8.38)	–	–	− 6.29 (− 30.98 | − 8.38)	–
Disease duration after onset (years)	–	–	6.53 (2.1 | 11.17)	–	–	7.54 (3.31 | 17.34)
SARA score	0 (0–2)	0 (0–2.5)	7.5 (3–16) ***			

Median and range (min–max) were reported. Significant differences: *p < 0.05; **p < 0.01; ***p < 0.001

### Cross-sectional evaluations at baseline

In the cross-sectional analysis at baseline, a significant difference was observed between CTRL participants and preSCA1 subjects in VPL nucleus (up to − 11%). Congruently, a significant decrease in the bilateral VLP nucleus volume (up to − 12%) was also observed in the comparison between CTRL participants and SCA1 patients, to whom a significant atrophy is extended to anterior, lateral, ventral, and intralaminar portions of thalamus and mediodorsal nuclei (up to − 56%) were added (Table 2).

**Table 2:** Thalamic nuclei volumes of CTRL, preSCA1, SCA1, preSCA2, and SCA2 subjects at baseline visits

Structural grouping	Thalamic Nuclei	CTRL	preSCA1	SCA1	PreSCA2	SCA2
		N = 20	N = 14	N = 10	N = 13	N = 13
		Median (Q1–Q3)	Median (Q1–Q3)	Median (Q1–Q3)	Median (Q1–Q3)	Median (Q1–Q3)
			*p [%*Δ* vs. CTRL]*	*p [%*Δ* vs. CTRL]*	*p [%*Δ* vs. CTRL]*	*p [%*Δ* vs. CTRL]*
Anterior	Anteroventral (AV)	0.018 [0.017–0.020]	0.018 [0.016–0.019]	0.015 [0.013–0.016]	0.019 [0.017–0.021]	0.016 [0.015–0.017]
			0.243 [− 1.03]	**0.001 [**− 19.06] *	0.756 [6.04]	**0.013 [**− 10.73] *
Lateral	Laterodorsal (LD)	0.003 [0.003–0.004]	0.003 [0.002–0.004]	0.001 [0.001–0.003]	0.004 [0.004–0.005]	0.003 [0.002–0.003]
			0.376 [− 4.37]	**0.002 [**− 56.54] *	0.396 [20.93]	**0.011 [**− 21.78] *
	Lateral Posterior (LP)	0.017 [0.015–0.019]	0.016 [0.015–0.018]	0.013 [0.010–0.016]	0.018 [0.017–0.019]	0.016 [0.012–0.017]
			0.315 [− 6.90]	**0.004 [**− 24.99] *	0.260 [10.52]	**0.031 [**− **3.26]**
Ventral	Ventral Anterior (VA)	0.051 [0.050–0.055]	0.051 [0.049–0.054]	0.046 [0.042–0.049]	0.055 [0.051–0.059]	0.047 [0.046–0.050]
			0.349 [− 0.06]	**0.001 [**− 10.59] *	0.707 [7.90]	**0.039 [**− **8.48]**
	Ventral Lateral anterior (VLa)	0.085 [0.084–0.090]	0.081 [0.077–0.085]	0.072 [0.066–0.077]	0.091 [0.079–0.099]	0.076 [0.075–0.082]
			0.126 [− 4.69]	**0.000 [**− 15.19] *	0.734 [6.60]	**0.011 [**− 11.10] *
	Ventral Lateral posterior (VLp)	0.116 [0.110–0.126]	0.106 [0.102–0.111]	0.096 [0.087–0.102]	0.119 [0.108–0.132]	0.101 [0.100–0.105]
			0.077 [− 8.46]	**0.000 [**− 17.08] *	0.670 [2.62]	**0.008 [**− 13.06]*
	Ventral Posterolateral (VPL)	0.128 [0.116–0.142]	0.114 [0.104–0.125]	0.112 [0.100–0.115]	0.131 [0.119–0.150]	0.111 [0.109–0.121]
			**0.023 [**− **10.92]**	**0.004 [**− 11.86] *	0.466 [3.08]	**0.030 [**− **12.74]**
	Ventromedial (VM)	0.004 [0.003–0.004]	0.003 [0.003–0.003]	0.003 [0.002–0.003]	0.004 [0.003–0.004]	0.003 [0.003–0.003]
			0.219 [− 9.03]	**0.015 [**− 24.87] *	0.161 [6.39]	**0.040 [**− **17.59]**
Intralaminar	Intralaminar	0.059 [0.057–0.064]	0.057 [0.053–0.061]	0.050 [0.045–0.054]	0.063 [0.057–0.068]	0.052 [0.051–0.054]
			0.144 [− 3.753]	**0.000 [**− 15.464] *	0.625 [6.584]	**0.002 [**− 11.651] *
Medial	Midline	0.003 [0.003–0.003]	0.003 [0.003–0.003]	0.002 [0.002–0.003]	0.003 [0.003–0.004]	0.003 [0.002–0.003]
			0.798 [− 7.966]	0.052 [− 19.357]	0.099 [2.917]	0.314 [− 13.933]
	Mediodorsal (MD)	0.133 [0.121–0.141]	0.117 [0.110–0.137]	0.107 [0.092–0.126]	0.131 [0.117–0.135]	0.112 [0.100–0.120]
			0.186 [− 11.804]	**0.002 [**− 19.351] *	0.659 [− 1.385]	**0.002 [**− 15.842] *
Posterior	Lateral Geniculate (LGN)	0.033 [0.031–0.037]	0.036 [0.032–0.038]	0.033 [0.028–0.035]	0.038 [0.034–0.042]	0.034 [0.031–0.037]
			0.339 [8.566]	0.229 [− 0.573]	**0.044 [16.73]**	0.883 [4.259]
	Medial Geniculate (MGN)	0.015 [0.013–0.017]	0.013 [0.011–0.016]	0.014 [0.014–0.015]	0.015 [0.014–0.017]	0.016 [0.014–0.017]
			0.184 [− 10.636]	0.851 [− 1.383]	0.311 [3.721]	0.564 [8.232]
	Pulvinar	0.223 [0.215–0.240]	0.229 [0.208–0.246]	0.216 [0.197–0.228]	0.255 [0.225–0.270]	0.233 [0.221–0.244]
			0.944 [2.441]	0.053 [− 3.307]	**0.013 [14.095]**	0.657 [4.191]

Median, 1st quantile (Q1), and 3rd quantile (Q3) of nuclei volume (left and right hemisphere sum) for each group are reported. Multinomial logistic regression was used to assess significant differences in volume among participants groups. Statistically significant values are in bold and increase (+) or decrease (−) of volume in median percentage variations from CTRL is reported. (*) Significant values after correction for multiple comparisons (*p* < 0.05 FDR)

A significant increase in the pulvinar and LGN volumes (up to 17%) was reported in preSCA2 when compared with CTRL subjects. Considering SCA2 patients compared to CTRL subjects, a significant decrease in volume was found in the anterior, lateral, ventral, and intralaminar portions of thalamus and mediodorsal nuclei (up to − 22%) (Table 2).

### Longitudinal evaluation

#### preSCA1/SCA1

**Fig. 1:**
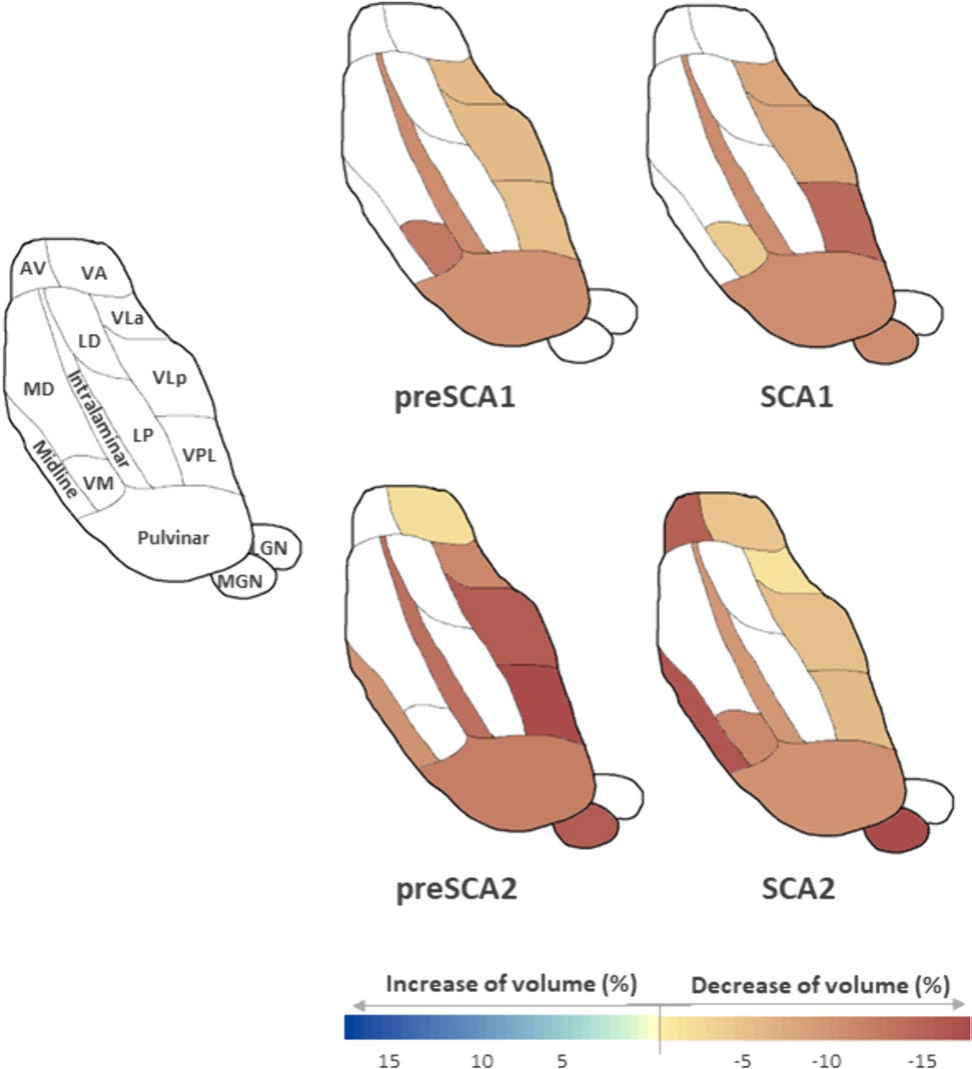
Longitudinal thalamic nuclei volume changes in preSCA1, SCA1, preSCA2, SCA2 subjects. Percentage of the median variation between baseline and 1-year follow-up visit within each group is reported only in the thalamic nuclei that presented significant alterations in the Wilcoxon signed rank test. The cartoon, adapted from [38], is a schematic representation of an axial view of the thalamic nuclei

In the longitudinal evaluation, preSCA1 subjects showed a significant decrease of the volume in the ventral portion of the thalamus (up to − 13%) including VLa, VLp, VPL, and VM, the intralaminar (up to − 11%), and pulvinar (up to − 9%) nuclei (Table 3, Fig. 1).

**Table 3:** Longitudinal thalamic nuclei volume changes

Groups		AV	LD	LP	VA	VLa	VLp	VPL	VM	Intralaminar	Midline	MD	LGN	MGN	Pulvinar
PreSCA1	%	–	–	–	–	− 7.79	− 7.47	− 5.35	− 12.65	− 10.84	–	–	–	–	− 9.16
N = 8	*p* value	n.s	n.s	n.s	n.s	0.016	0.023	0.016	0.039	0.008	n.s	n.s	n.s	n.s	0.023
SCA1	%	–	–	–	–	− **7.72**	− **7.55**	− **13.3**	− **4.13**	− **10.06**	–	–	–	− 10.32	− **10.1**
N = 9	*p* value	n.s	n.s	n.s	n.s	0.008 *	0.012 *	0.004 *	0.008 *	0.020 *	n.s	n.s	n.s	0.039	0.004 *
PreSCA2	%	–	–	–	− 2.73	− **11.87**	− **15.56**	− **20.13**	–	− **13.17**	− **9.1**	–	–	− **16.62**	− **12.92**
N = 9	*p* value	n.s	n.s	n.s	0.039	0.020 *	0.020 *	0.004 *	n.s	0.004 *	0.020 *	n.s	n.s	0.004 *	0.004 *
SCA2	%	− **14.04**	–	–	− **4.57**	− **2.17**	− **5.78**	− **6.09**	− **11.32**	− **8.62**	− **16.4**	–	− **7.78**	− **21.28**	− **9.9**
N = 13	*p* value	0.013 *	n.s	n.s	0.027 *	0.000 *	0.001*	0.001 *	0.000 *	0.001 *	0.000 *	n.s	0.013 *	0.001 *	0.000 *

Wilcoxon signed rank test was used to observe the longitudinal effect intra-group. For an exploratory analysis, statistically significant values (*p* < 0.05) were reported. In bold significant values after correction for multiple comparisons (*p* < 0.05 FDR) were highlighted. For each significant thalamic nucleus, decrease (−) of volume in median percentage change between baseline and 1-year follow-up visit within each group (%) is reported. Abbreviations: n.s. = not significant

In SCA1 patients, the nuclei that were longitudinally altered in the preSCA1 patients also showed a significant decrease in volume (i.e. VLa, VLp, VPL, intralaminar, and pulvinar, up to − 13%). In addition, further alterations were reported in MGN (up to − 10%) nuclei (Table 3, Fig. 1). The volumes of the VPL, VLa, and VLp nuclei shown were reduced with effect sizes ranging from very large to huge, both in preSCA1 and SCA1 patients (preSCA1 − 1.3; SCA1 − 1.5).

#### preSCA2/SCA2

In the longitudinal evaluation, preSCA2 subjects reported significant thalamic changes in the ventral portion of the thalamus (up to − 20%) including VLa, VLp, VPL, and VA, the intralaminar (up to − 13%), the midline (up to − 9%), and the posterior portion of thalamus (up to − 17%) including MGN and pulvinar nuclei (Table 3, Fig. 1).

In SCA2 patients, a significant volume decrease was observed in the same nuclei reported as significant in preSCA2 subjects (VLa, VLp, VLP, VA, intralaminar, midline, MGN, and pulvinar, up to − 21%). Moreover, SCA2 patients showed significant longitudinal volume changes in the AV (up to − 14%), VM (up to − 16%), and LGN (up to − 11%) (Table 3, Fig. 1). VLP, Vla, and VLp nuclei showed very large to huge changes in the effect size (preSCA1 − 1.4; SCA1 − 1.8).

### Correlations

The volume of the ventral portion including VPL, Vla, and VLp nuclei revealed significant associations with SARA score (*p* < 0.01) and time of disease progression (*p* < 0.008) (Table 4). Moreover, the volume of this thalamic portion was also correlated with total cerebellar volume (*p* < 0.04). Additionally, a significant correlation was found between the volume of this region and the volumes of specific cerebellar lobules comprising IV, V, VI, Crus II, VIIB, VIIIA, VIIIB for preSCA1 and SCA1 (*p* < 0.012) and I–II, IV, VI in preSCA2 and SCA2 (*p* < 0.037).

**Table 4:** Correlations

VLa VLp VPL	preSCA1–SCA1		preSCA2–SCA2	
	estimate	*p* value	estimate	*p* value
SARA	− 0.52	**0.009**	− 0.5	**0.010**
Time of disease progression	− 0.58	**0.003**	− 0.51	**0.008**
Age	− 0.33	0.115	0.14	0.489
Total cerebellum	0.49	**0.014**	0.4	**0.044**
Lobule IV V VI VIIB VIIIA VIIIB Crus II	0.5	**0.012**	–	–
Lobule I II IV VI	–	–	0.41	**0.037**

Correlations of the baseline volume of the thalamic portion including the VPL, VLa, VLp nuclei with clinical variables and cerebellar volume. In bold significant values (*p* < 0.05) were highlighted. For presymptomatic individuals, the estimated years-to-onset were considered

## Discussion

Characteristic MRI features associated with SCA1 and SCA2 genotypes had already investigated in several studies. Volume changes in pons and cerebellar vermis and hemispheres were already detected in ataxic patients and in presymptomatic individuals [7–15]. We previously showed in the same series of patients that cerebellar atrophy can be detected in SCA1 and SCA2 subjects approximately a decade before symptom or signs of the disease become manifest [14, 15].

Here, we showed that these patients also have early thalamic alterations. A similar pattern of atrophy extending throughout the ventral, anterior, lateral, intralaminar, and medial portions of the thalamus was observed in both SCA1 and SCA2 patients as early as the pre-manifest stages. The longitudinal alterations mainly involved the ventral portion of the thalamus, including the VPL, VLa, and VLp nuclei, as well as its intralaminar and posterior portions.

Overall, our results support the hypothesis that the different thalamus nuclei are involved in different stages of the degenerative process in SCA1 and SCA2 [18].

In presymptomatic individuals, longitudinal evaluation at 1 year showed a specific pattern of thalamic nuclei atrophy in the ventral portion of the thalamus—mainly the VPL, VLa, and VLp—and intralaminar nuclei, whereas in symptomatic individual longitudinal volume decreases were also evident in the remaining ventral portions (VA and/or VM).

Furthermore, we have shown that the volume of the ventral thalamic portion showed a significant correlation with both SARA and time of disease progression, as well as with the total cerebellar volume and the most atrophic lobules, underlining the close link with disease progression.

Based on pathoanatomical studies [16–18], the ventral nuclei of the thalamus, mainly VPL, VLa, and VLp, were consistently reported to be the most common targets of the degenerative process in both SCA1 [16] and SCA2 [17, 18]. The thalamic VPL, VLa, VLp, and centromedian intralaminar nuclei are closely related to motor processes [42, 43]. The whole ventral portion of the thalamus has strong connections with the deep cerebellar nuclei, particularly the dentate nucleus, as well as the internal globus pallidum [32]. These early patterns of alteration in SCA1 and SCA2 support the hypothesis that the thalamus, especially ventral, plays a central role in the degeneration of the cerebellum-thalamus-cortical and basal ganglia-thalamus-cortical pathways (core neural somatomotor loops) from the presymptomatic phase of the disease [22].

Moreover, according to our observations, preSCA1 subjects, when compared to healthy participants, showed an early degeneration of the VPL nucleus—the thalamic nucleus with the largest number of connections in the dentate-thalamic pathway [32, 43]. Notably, VPL is involved in sensory processing by transmitting tactile, proprioceptive, and nociceptive signals to the somatosensory cortex [44]. These ventral thalamic longitudinal alterations may be associated with sensorimotor deficits, peripheral neuropathy, and motor performance decline detected in presymptomatic subjects, years before clinical onset [2, 4, 22, 45, 46].

The volume of pulvinar was also found to decrease at longitudinal evaluation. It is a higher-order structure associated with visuospatial integrations, such as saccadic dysfunction and impaired visual attention [47]. In line with our findings, neuropathological studies have reported damage in the inferior and lateral pulvinar regions in several patients with SCA2 [18] and in visual nuclei in patients with SCA1 [16]. This nucleus’s longitudinal alteration may be associated with the oculomotor dysfunction described in both SCA1 and SCA2 patients [22, 48]. In addition, our cross-sectional analysis revealed that preSCA2 individuals—farther from disease onset than preSCA1 (median 16 vs. 9 years)—had a larger pulvinar and LGN volume compared to controls. The increased volume in these thalamic nuclei could be related to compensatory effects that may precede structural degeneration [46]. Indeed, several studies on preSCA2 report that alterations in saccadic movements are among the first dysfunctions to become apparent years before symptoms begin [45, 46, 49].

Other nuclei, such as MGN, midline, and AV, exhibit longitudinal alterations mainly in the presymptomatic and manifest SCA2 patients and could be associated with early alterations in sensory, limbic, and executive functions [46, 50].

Finally, cross-sectional data in both SCA1 and SCA2 patients confirmed an important widespread atrophy extending to almost the entire thalamus except for its posterior and partly medial portion in the symptomatic phase of the disease. The observed volume decreases are consistent with grey matter alterations detected by voxel-based morphometry in symptomatic patients [25, 26, 28]. Such widespread alterations could be related to the development of the cognitive motor dysfunctions described in the symptomatic phase [51].

This study has, however, some limitations. We used an automated method to derive the volume of thalamic nuclei, instead of the manual segmentation. Our tool outperformed human inter-rater accuracy levels, reaching intra-rater precision, and being resistant to MRI contrast changes. Furthermore, the 52 bilateral thalamic regions were combined into 14 regions in order to further reduce the residual effects of less reliable segmentation on T1-weighted MRI.

## Conclusion

Our results showed for the first time *in vivo* that different thalamic nuclei are involved at different stages of the degenerative process. Atrophy of specific thalamic nuclei, mainly ventral and occipital, might play a significant role in the progression of disease years before clinical onset in both SCA1 and SCA2. Further longitudinal studies should be performed to confirm thalamic atrophy as one of the earliest event in the pathophysiology of SCA 1 and SCA2 diseases.

### Supplementary Information

Below is the link to the electronic supplementary material.

Supplementary file1 (DOCX 21 KB)

## Data Availability

The data will be available upon reasonable request to the corresponding author. Dr Anna Nigri had full access to all of the data in the study and took responsibility for the integrity of the data and the accuracy of the data analysis.

## Abbreviations

SCA1: Spinocerebellar ataxia type 1
SCA2: Spinocerebellar ataxia type 2
preSCA1: Presymptomatic SCA1 mutation carriers
preSCA2: Presymptomatic SCA2 mutation carriers
CTRL: Healthy participants
SARA: Scale for Assessment and Rating of Ataxia
T1w: T1-weighted
TIV: Total intracranial volume
AV: Anteroventral nucleus
LD: Laterodorsal nucleus
LP: Lateroposterior nucleus
VA: Anterior ventral nucleus
Vla: Anterior lateral ventral nucleus
VLp: Posterior lateral ventral nucleus
VPL: Posterolateral ventral nucleus
VM: Ventromedial nucleus
MD: Mediodorsal nucleus
LGN: Lateral geniculate nucleus
MGN: Medial geniculate nucleus
