# Supplementary material for: Tracking longitudinal thalamic volume changes during early stages of SCA1 and SCA2
Source: Radiol Med. 2024 Jul 2;129(8):1215–23. doi: 10.1007/s11547-024-01839-2 (PMC11322486; doi:10.1007/s11547-024-01839-2)
Supplement: Supplementary file 1 — Supplementary file1 (DOCX 21 KB) [file 11547_2024_1839_MOESM1_ESM.docx]

**Supplementary materials**

**Supplementary Table 1.** The subgroups of the subnuclei of the thalamus reported in the study versus the classification of the subnuclei obtained through Freeserfer's segmentation are shown. Such subgrouping was carried out as reported in Bocchetta et al. 2020.

| **Included?** | **subgroup** | **Freesurfer** |
| --- | --- | --- |
| Yes | AV | AV |
| Yes | LD | LD |
| Yes | LP | LP |
| Yes | VA | VA |
| Yes |  | VAmc |
| Yes | VLa | VLa |
| Yes | VLp | VLp |
| Yes | VPL | VPL |
| Yes | VM | VM |
| Yes | Intralaminar | CeM |
| Yes |  | CL |
| Yes |  | Pc |
| Yes |  | CM |
| Yes |  | Pf |
| Yes | Midline | Pt |
| Yes |  | MV-re |
| Yes | MD | MDm |
| Yes |  | MDl |
| Yes | LGN | LGN |
| Yes | MGN | MGN |
| Yes | Pulvinar | PuA |
| Yes |  | PuM |
| Yes |  | PuL |
| Yes |  | PuI |
| No |  | R |
| No |  | L-SG |
